# Oral Diseases as a Manifestation of Inborn Errors of Immunity

**DOI:** 10.3390/jcm13175079

**Published:** 2024-08-27

**Authors:** Katarzyna Napiórkowska-Baran, Samira Darwish, Justyna Kaczor, Paweł Treichel, Bartłomiej Szymczak, Maciej Szota, Kinga Koperska, Zbigniew Bartuzi

**Affiliations:** 1 Department of Allergology, Clinical Immunology and Internal Diseases, Collegium Medicum Bydgoszcz, Nicolaus Copernicus University Torun, 85-067 Bydgoszcz, Poland; zbartuzi@cm.umk.pl; 2 Student Research Club of Clinical Immunology, Department of Allergology, Clinical Immunology and Internal Diseases, Collegium Medicum Bydgoszcz, Nicolaus Copernicus University Torun, 85-067 Bydgoszcz, Poland; samiradarwish00@gmail.com (S.D.); justyna.kaczor.98@gmail.com (J.K.); treichel.pawel@gmail.com (P.T.); bartlomiej.szymczak1@gmail.com (B.S.); 77maciek77@gmail.com (M.S.); kingakoperska@gmail.com (K.K.)

**Keywords:** inborn errors of immunity, primary immunodeficiencies, immune defects, oral infection, oral diseases, oral pathology

## Abstract

Oral findings such as inflammation, ulcerations, or lesions can indicate serious systemic diseases and should prompt suspicion of acquired chronic conditions or inborn errors of immunity (IEIs). Currently, there are approximately 500 disease entities classified as IEIs, with the list expanding annually. The awareness of the existence of such conditions is of paramount importance, as patients with these disorders frequently necessitate the utilization of enhanced diagnostic techniques. This is exemplified by patients with impaired antibody production, in whom conventional serological methods may prove to be undiagnostic. Patients with IEI may require distinct therapeutic approaches or antimicrobial prophylaxis throughout their lives. An accurate diagnosis and, more importantly, early identification of patients with immune deficiencies is crucial to ensure the quality and longevity of their lives. It is important to note that the failure to establish a proper diagnosis or to provide adequate treatment could also have legal implications for medical professionals. The article presents IEIs, which may manifest in the oral cavity, and their diagnosis alongside therapeutic procedures.

## 1. Introduction

The oral cavity plays an important role in general health, including digestive, respiratory, articulatory, and protective functions. In the context of respiratory disorders, such as nasal obstruction, it represents the initial point of contact with pathogens. Changes in the oral cavity, such as inflammation, ulcerations, or oral lesions, may not only result in local pathology but also contribute to the development of more serious infections. Such conditions may result from the acquired chronic diseases, but they may also indicate inborn errors of immunity (IEIs). Oral diseases encompass all pathological changes affecting the gums, tongue, soft and hard palate, and mucous membranes. The ramifications of these alterations may be mild and transient. In some cases, complications may develop, such as infections of the upper and lower respiratory tract, heart disease (including endocarditis), or systemic infection.

Oral diseases encompass a broad group of conditions that can affect both the soft and hard tissues of the oral cavity. These diseases may be associated with craniofacial abnormalities, injuries, infectious diseases, and congenital defects [1,2]. The World Health Organization identifies the following oral diseases as major public health challenges: dental caries, periodontal diseases, complete tooth loss, and oral cancers [3].

**Figure 1:**
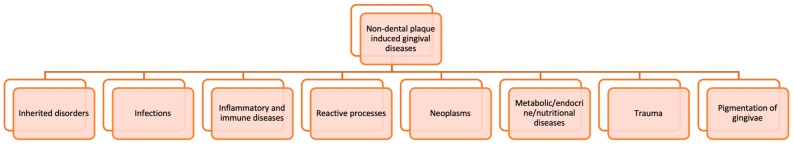
Classification of non-dental plaque induced gingival diseases [7].

According to the Global Estimates of Burden of Oral Conditions in 2017, the prevalence of all oral diseases exceeded 3.4 billion cases. This included over 500 million cases of dental caries in primary teeth, more than 2 billion cases of dental caries in permanent teeth, over 700 million cases of severe periodontal disease, more than 250 million cases of complete tooth loss, and over 130 million cases of other periodontal diseases. A significant increase in the prevalence of all oral diseases has been observed in developing countries, while a decrease has been noted in some developed countries [4]. Figure 1 shows the important classification of non-dental plaque induced gingival diseases.

Periodontal diseases are a special form of oral diseases. This is a group of diseases that involve the inflammation of the tissues surrounding the tooth. The inflammatory changes can affect the gums as well as connective tissue, ligaments, and bone, which are the tissues that support the tooth [5,6]. The natural course of periodontal disease begins with gingivitis, which is a reversible condition. Prolonged inflammation leads to inflammation of deeper tissues and the development of periodontitis [5,7].

Moreover, oral cancers rank 13th among the most common cancers. This group of diseases includes malignant neoplasms affecting the oral cavity, lips, and pharynx [3,8]. According to the World Health Organization’s International Agency for Research on Cancer, there were 389,846 cases of oral cancer globally in 2022, with nearly 270,000 cases occurring in men. The number of deaths due to oral cancer in 2022 was 188,438 [8].

The primary etiological factors associated with oral cancer include tobacco use, alcohol consumption, and betel quid chewing. Studies indicate that low intake of vegetables and fruits also increases the risk of this type of cancer [9,10]. Oral cancer can also result from viral and fungal infections, immunosuppression, and genetic factors [10,11,12,13]. The most common type of oral cancer is squamous cell carcinoma [10,14]. Due to the nonspecific and late-appearing symptoms, oral cancer poses a significant challenge for early detection and appropriate treatment [15,16].

The article provides a concise overview of IEIs that can manifest with oral lesions, diagnostic procedures, and therapeutic management options. 

## 2. Oral Diseases as a Manifestation of Inborn Errors of Immunity

### 2.1. Oral Cavity as a Part of the Mucosal Immune System

The oral cavity is covered by a mucous membrane, playing a crucial role as a protective barrier against pathogens and antigens, thanks to the cooperation of the innate and acquired immune systems. The oral epithelium, although multilayered, shows diverse adaptations depending on the function in different areas, such as the periodontal environment, which is particularly rich in a diverse bacterial flora. The oral cavity, hosting over 700 microbial species, has the second largest and most diverse microbiota after the gut. This complex microbiota, which evolves to support oral health, maintains a dynamic balance with the host, colonizing both the hard surfaces of teeth and the soft tissues of the oral mucosa. Commensal microbiota are critical regulators of barrier immune function, shaping protective and homeostatic immune responses at mucosal surfaces. However, the formation of complex bacterial biofilms, which are recognized as virulence factors in various oral infectious diseases, can compromise epithelial barrier integrity. These biofilms, comprising diverse microbial communities embedded in a matrix of bacterial and salivary polymers, can disrupt barrier function by modulating barrier-associated genes and proteins or by directly compromising junctional integrity, facilitating pathogen access to underlying tissues [17,18,19,20,21,22]. Physical barriers, such as tight junctions between epithelial cells and the mucin layer covering them, along with biochemical barriers, including antimicrobial peptides, form the first line of defense. The epithelium is anchored to the underlying extracellular matrix by a fibrous basement membrane with strong integrin bonds. Its structural integrity, maintained by tight, gap, and anchoring junctions, forms physical barriers that protect against environmental and microbial irritants. These cell–cell interactions are vital for the epithelium’s physiological processes and can quickly adapt to changing physiological and pathological conditions [18]. An important defensive element is saliva and gingival fluid. They contain secretory immunoglobulin A (SIgA), as well as many enzymes that neutralize bacteria and viruses [23,24]. Specific mechanisms have three main tasks: homeostatic, defensive, and regulatory. These specific mechanisms are managed by the mucosal immune system, which morphologically consists of tonsils, Peyer’s patches, solitary lymphoid follicles, and hematopoietic-derived cells such as macrophages, monocytes, dendritic cells, and B and T lymphocytes, with a predominance of CD4+ T lymphocytes over CD8+ T lymphocytes. This predominance makes it relatively difficult for inflammation to develop in the oral cavity. For instance, IL-17 produced by CD3+ CD4+ CD44hiTCRβ + CCR6+ natural Th17 (nTh17) cells and tongue-resident populations of γδ T cells promotes granulopoiesis and neutrophil accumulation in peripheral tissues for pathogen clearance [17]. In acquired immunity, SIgA, secreted by plasma cells located in the lamina propria, plays a crucial role by neutralizing pathogens and their toxins while also supporting the homeostasis of the mucous membrane. SIgA also coats the physiological bacterial flora of the oral cavity, preventing its excessive expansion and contact with endothelial cells [17,18,19,20]. Despite constant exposure to potentially pathogenic microorganisms, symptomatic infections in the oral mucosa are relatively rare, likely due to its ability to immunologically tolerate these microbes. This peripheral immune tolerance arises from either T cell anergy or suppression by regulatory T cells (T-reg). While mature dendritic cells activate strong adaptive immune responses, immature dendritic cells promote immune tolerance through low-level responses or anergy mediated by T-reg cells [20,21,22].

### 2.2. Inborn Errors of Immunity

Inborn errors of immunity, formerly known as primary immunodeficiencies, are a group of disorders that are characterized by an increased risk of developing autoimmune diseases, infections, allergies, or cancers. From 1980 to 2022, 485 genetic defects that are responsible for the occurrence of inborn errors of immunity have been identified. In 2022, the International Union of Immunological Societies Expert Committee (IUIS) recommended to classify inborn errors of immunity into 10 groups, each of which distinguishes disease types and subtypes, taking into account the phenotypic characteristics of the disease [25,26,27,28]. The classification of each IEI and its subtypes is presented in Table 1.

In 2018, the Jeffrey Modell Foundation proposed a list of 10 symptoms that may indicate primary immunodeficiency in both children and adults. The aforementioned symptoms are presented in Table 2 [29].

### 2.3. Inborn Errors of Immunity and Oral Diseases

As already mentioned, one of the symptoms that may indicate the presence of congenital defects of immunity are diseases and ailments of the oral cavity [28,29]. Table 3 presents a list of congenital immune defects that may include pathologies in the oral cavity, along with a brief description of co-occurring pathologies.

## 3. Characteristics of Selected IEI Associated with Periodontal Disorders

### 3.1. Immunodeficiencies Affecting Cellular and Humoral Immunity

Severe combined immunodeficiency (SCID) encompasses both cellular and humoral immunity. They are defined as the absence or a significant deficiency of functionally active T lymphocytes, which may be accompanied by a deficiency of B lymphocytes and/or natural killer (NK) cells. Among these, few subtypes might be distinguished: SCID with T lymphopenia and a conserved population of B lymphocytes (T-B+SCID; 10 disease entities), SCID with T and B lymphopenia (T-B-SCID; eight disease entities), and combined immunodeficiencies other than SCID (40 disease entities). In both SCID subtypes, the number of NK cells can be either normal (NK+) or reduced (NK−). The only exception is Omenn syndrome, where no significant T-cell deficiency is typically observed. Although combined immunodeficiencies are rare, they are being described with increasing frequency as diagnostic methods improve and screening programs are introduced. In the United States, the incidence of SCID is currently estimated at 1/58,000 live-born infants [102,103,104].

SCID symptoms include recurrent or chronic infections that do not respond to conventional treatment. These symptoms typically manifest within the first eight months of life, predominantly in the form of recurrent respiratory viral infections, chronic diarrhea-related weight and length increment disorders, severe bacterial infections (pneumonia, sepsis), and fungal infections. The administration of vaccines containing live microorganisms can result in the development of generalized infections, while transfusion of non-irradiated blood products can lead to the development of graft versus host disease (GvHD). SCID is a life-threatening condition, with an estimated life expectancy for untreated children of less than 2 years. In the case of combined immunodeficiencies with a milder course (e.g., associated with the hypomorphic mutations), clinical symptoms in the form of recurrent infections may first emerge in children aged 3–8 years. The treatment of choice for patients with SCID is hematopoietic cell transplantation (HCT), which should be performed as soon as possible because, as mentioned earlier, untreated CVID significantly increases mortality [102,105].

Lesions in the oral cavity subsume a spectrum of conditions, such as candidiasis, viral infections, and ulcerative stomatitis. Oral manifestations may also include severe necrotizing gingivostomatitis. However, periodontal disease associated with SCID is peculiar due to stem cell transplantation at a young age and is often the result of complications following this procedure [106,107].

### 3.2. Combined Immunodeficiencies with Associated or Syndromic Features

#### 3.2.1. Wiskott–Aldrich Syndrome (WAS)

Wiskott–Aldrich syndrome is an inherited disorder that follows an X-linked pattern of inheritance. It is associated with mutations in the WAS gene encoding the WASP protein, whose expression is found in cells of all hematopoietic lineages (CD34-positive stem cells, platelets and lymphocytes, including NK cells). Disruption of WASP synthesis interferes with signal transduction in hematopoietic cells and reorganization of the cytoskeleton through regulation of actin polymerization. The incidence of WAS in the United States is approximately 1 case per 250,000 births. WAS is characterized by microthrombocytopenia, eczema, recurrent infections, and an increased incidence of autoimmunity and malignancies (the most frequent malignancy is an Epstein–Barr virus (EBV)-positive B-cell lymphoma) [46,108].

The periodontal symptoms of this syndrome include gingival ulceration with a bleeding tendency and palatal petechiae [108]. Periodontitis and infections may result from bleeding gums. Thrombocytopenia, which is a feature of WAS, can cause periodontitis by damaging the continuity of the mucosa, thereby allowing bacteria to invade and facilitating their proliferation in the tissues. On the other hand, periodontitis causes increased fragility of the mucosa, making it more susceptible to bleeding gums. Moreover, individuals diagnosed with WAS syndrome are more prone to develop aphthae than the general population [109].

Currently, the treatment of WAS involves the use of symptomatic treatment in conjunction with prophylaxis against infection. Nevertheless, hematopoietic stem cell transplantation and/or lentivirus gene therapy have been studied and described as promising approaches [110].

#### 3.2.2. DiGeorge Syndrome (DGS)

DiGeorge syndrome is associated with a 22q11 deletion that results in impaired embryogenesis of organs derived from the third and fourth pharyngeal arches. Due to the defects present in this syndrome, DGS is otherwise known by the acronym CATCH22, which stands for the following defects: cardiac abnormalities, abnormal facies, thymic hypoplasia, cleft palate, and hypocalcemia. Patients with DGS are distinguished by a dysmorphic facial appearance with hypertelorism, low and set-back auricles, a short fossa, and a thick and reflective mouth, as well as micrognathia. Craniofacial defects may also include hypoplasia of the oral portion of the pharynx, cleft or arched palate. The prevalence of DGS in the general population is estimated to be 1:4000 [38,107].

A number of dental abnormalities, including enamel hypoplasia, missing teeth, and dental caries, have been observed in DGS. It has been demonstrated that enamel defects are more frequently associated with hypomineralization than hypoplasia [111,112].

Improving the quality of life of patients affected with this syndrome may be viable with the implementation of early diagnosis and treatment modalities that target the affected organ systems. In complete DGS, allogeneic heterotopic thymus tissue transplantation is performed. In some patients, immunoglobulin replacement therapy may be beneficial. Antimicrobial prophylaxis is not typically required for patients with this condition [113].

#### 3.2.3. Ataxia–Telangiectasia (A–T)

Ataxia–telangiectasia is a genetic defect of the DNA repair mechanism in which the mutation involves the ATM protein kinase gene mapped on chromosome 11q. This enzyme activates other proteins responsible for cell cycle regulation, DNA damage repair, and apoptosis. A mutation of this gene renders cells highly susceptible to radiation-induced chromosomal damage. The prevalence of A–T in the United States is estimated to be approximately 1 in 40,000 individuals. The disease has a particularly deleterious effect on the nervous and immune systems, and patients with conditions related to those systems have a greater predisposition to develop malignant tumors. Patients exhibit impaired motor coordination (ataxia), ocular telangiectasias, frequent infections (especially pulmonary), and an increased incidence of leukemia and lymphoma. A progressive decrease in the number of T lymphocytes and a frequent decrease in IgA, IgE, and IgG are observed. A higher prevalence of recurrent herpetic gingivitis and candidiasis has been reported in patients with A–T. The diagnosis of A–T is based on molecular diagnostics, following the identification of pathogenic variants of the ATM gene. The diagnosis of A–T is typically based on the results of sequencing the ATM gene. An Immunoblot test may also be performed, with the absence or reduced amount of ATM protein serving to support the diagnosis. The treatment of this condition is primarily symptomatic. Neurological deterioration is progressive and currently no causal treatment for this disease is available. It is recommended that radiological examinations be performed with the utmost caution and as sparingly as possible in order to ensure the protection of individuals from ultraviolet radiation [28,114,115].

#### 3.2.4. Hyper-IgE Syndrome (HIES)

Hyper-IgE syndromes are a group of genetic diseases characterized by elevated IgE expression. Two forms of HIES can be distinguished: autosomal dominant (AD) inherited HIES (also known as Job’s syndrome) and several autosomal recessive (AR) forms of HIES. The incidence of HIES is difficult to determine, given the small number of cases described. Some sources estimate that HIES occurs at a frequency of 1:1,000,000. AD-HIES is caused by mutations in the STAT3 protein gene, while AR-HIES is associated with mutations in the TYK2 gene and the DOCK8 gene [116].

The STAT3 protein belongs to a family of signal transducers and activators of transcription that play essential roles in fetal development, wound healing, angiogenesis, immune function, and tumorigenesis. STAT3 plays a pivotal role in the secretion or signaling of numerous cytokines, including IL-6, IL-10, IL-11, IL-17, IL-21, IL-22, IL-23, leukemia inhibitory factor, oncostatin M, cardiotrophin-1, a cardiotrophin-like cytokine, and ciliary neurotrophic factor. In patients with AD-HIES, a reduction in the number of CD4 helper T cells (Th17) is observed. In contrast, in patients with AR-HIES a reduction in the number of T cells (with a more significant proportion of CD8 than CD4) was accompanied by a normal or reduced B cell count. The most typical symptoms include eczema, skin abscesses, respiratory tract infections, mucocutaneous candidiasis, and eosinophilia. Individuals diagnosed with AD-HIES exhibit distinctive abnormalities in their dentition, skeletal system and connective tissue, in contrast to those diagnosed with AR-HIES. Oral-related manifestations in patients with HIES include ligneous gingivitis/periodontitis, retained primary teeth, arched palate, and recurrent fungal infection [116]. The presence of characteristic symptoms and elevated serum IgE levels serve as the foundation for the diagnosis of HIES. A scoring scale is helpful for establishing the diagnosis [117]. Patients with HIES demonstrate serum IgE levels well above 2000 IU/mL, typically exceeding the upper range of normal (or 95th percentile) by at least 10-fold. In addition, eosinophilia usually presents with values exceeding 700 cells per μL. The treatment of HIES involves administration of antibiotic and antifungal medicaments, in order to prevent and treat emerging or ongoing infections. It is also necessary to treat eczema and to drain abscesses that form [118].

#### 3.2.5. Schimke’s Immuno-Osseous Dysplasia (SIOD)

SIOD is a rare disease with an estimated incidence of less than one in one million, inherited in an autosomal recessive manner. The disease is caused by a mutation in the SMARCAL1 gene, which encodes a protein involved in chromatin binding and remodeling. The prognosis and severity of symptoms associated with SIOD depend on the specific variant mutation of the SMARCAL1 gene. The most common and earliest symptoms are renal failure and proteinuric nephropathy [119].

Patients with SIOD are also characterized by reduced T-lymphocyte count, recurrent lymphopenia, spondyloepiphyseal dysplasia (SED) and facial dysmorphia. SIOD is associated with a number of dental anomalies, including microdontia, hypodontia, short and thin tooth roots, and misshapen primary and/or permanent molars (bulbous molar crowns). Regardless of the severity of the disease, patients with SIOD were diagnosed with caries more often than individuals in the healthy population. The treatment of patients is mainly symptomatic, as there is currently no therapy that can cure patients. It is of great significance to correctly diagnose the condition, since glucocorticosteroids, which act as immunosuppressants, are frequently employed in the treatment of nephropathy in patients. This treatment option, however, is contraindicated in SIOD patients [120].

### 3.3. Predominantly Antibody Deficiencies

Primary immunodeficiencies with predominantly antibody production disorders account for approximately 50–60% of all primary immunodeficiencies. The most severe course is seen in primary immunodeficiencies associated with decreased concentrations of all immunoglobulin classes (including X-linked agammaglobulinemia, formerly known as Bruton’s disease). The mildest clinical course is observed in SIgAD [121].

#### 3.3.1. Selective Immunoglobulin a Deficiency (SIgAD)

Selective immunoglobulin A deficiency (SIgAD) is the most prevalent primary antibody deficiency. It occurs with a frequency of approximately 1:500 among Caucasian populations. The pathogenesis of this condition remains unknown. However, it is believed to be related to defective differentiation of B cells, resulting in decreased IgA production. In over 50% of cases, the course of the disease is asymptomatic, with the remaining patients primarily experiencing increased susceptibility to infection and autoimmunity. Due to the protective role of secretory IgA on mucosal surfaces, individuals with IgA deficiency may be more susceptible to lung infections, allergies, autoimmune diseases, gastrointestinal disorders, malignancies, periodontal diseases, and other oral manifestations. Periodontitis or hyperplastic candidal infection may also occur. Children with SIgAD are particularly at risk of developing dental caries [107,122,123,124].

#### 3.3.2. Common Variable Immunodeficiency

Common variable immunodeficiency (CVID) is a group of congenital antibody deficiencies characterized by decreased concentrations of IgG and IgA or IgM and poor response to vaccination. CVID is diagnosed after the age of four, as the immune system matures by this time, and after exclusion of other immunodeficiencies (including cellular) and secondary hypogammaglobulinemia. The term “Variability” refers to the variation in symptoms and clinical course, which may include recurrent infections, signs of autoimmunity and increased risk of cancer. CVID occurs in both genders with a frequency of 1:10,000 to 1:50,000. It can manifest at any age, with the second to fourth decade of life being the most common period for diagnosis. A family history of CVID, isolated IgA deficiency (SIgAD) and transient hypogammaglobulinemia of early childhood is found in over 20% of cases. Depending on the clinical manifestations, patients with CVID can be divided into two groups. CVID may present solely as an infection or in conjunction with autoimmune, inflammatory, or lymphoproliferative manifestations. Recurrent infections occur in most patients, with the respiratory system being the most frequently affected (otitis media, sinusitis, bronchitis, and pneumonia). These infections are typically caused by enveloped bacteria, such as Haemophilus influenzae, Streptococcus pneumoniae, and Mycoplasma spp. Autoimmune diseases occur in about 20% of patients [125,126,127].

Oral lesions may include gingivitis, lichenoid lesions with Wickham striae, and necrotizing ulcerative periodontitis. It is possible to develop sepsis from odontogenic infection [107,128].

#### 3.3.3. Other Immunodeficiencies Involving Antibody Deficiency

Oral lesions have also been described in various humoral immune deficiencies as isolated IgG subclass deficiency (increased susceptibility to viral infections in IgG3 deficiency) and transient hypogammaglobulinemia of infancy (oral candidiasis) [128].

Isolated IgG subclass deficiency leads to increased frequency and severity of mucosal and systemic infections, especially respiratory infections. IgG is divided into four subclasses: IgG1, IgG2, IgG3, and IgG4. Low levels of one IgG subclass may, but do not have to, indicate a disease state. Interestingly, isolated deficiencies of IgG3 and IgG4 have not been conclusively confirmed. IgG2 deficiency increases susceptibility to recurrent pulmonary infections and the risk of bronchiectasis. It is more crucial to ascertain low levels of antibodies against specific bacterial antigens (Haemophilus influenzae type B, pneumococci, tetanus, and diphtheria), as this better identifies individuals at risk of infection [129,130,131].

Transient hypogammaglobulinemia of infancy (THI) is a primary heterozygous immune disorder characterized by reductions in both immunoglobulin G and immunoglobulin A. These reductions typically occur between the age of 5 and 24 months, with levels usually returning to normal between the ages of 2 and 6 years. The symptoms of THI include severe pneumonia, opportunistic fungal or Staphylococcus infections, gastrointestinal issues, and bloodstream infections. The diagnosis is typically made when IgG levels fall below two standard deviations from the mean for the patient’s age [132,133].

### 3.4. Diseases of Immune Dysregulation

Such a group of diseases is defined as having a defect of immune tolerance of their own antigens, leading to autoimmune disorders that may or may not be accompanied by recurrent infections. Selected diseases can progress with pathological changes in the oral cavity [28].

#### 3.4.1. Chediak–Higashi Syndrome

Chediak–Higashi syndrome (CHS) is a rare autosomal recessive disorder marked by congenital immunodeficiency, bleeding tendency, recurrent bacterial infections, partial albinism, and progressive neurodegeneration. Mutations in the lysosomal trafficking regulator (LYST) gene were identified to be causative of Chediak–Higashi, but despite many analyses, there is little functional information about the LYST protein. A crucial diagnostic feature of CHS is the presence of enlarged granules in leukocytes observed in a peripheral blood smear. Neutropenia, as well as decreased number and impaired function of NK cells, may also be present. Individuals with CHS may also experience aggressive periodontal inflammation, particularly those with atypical forms of the disease or those who have undergone hematopoietic stem cell transplantation [134,135,136].

However, it is currently unclear whether oral lesions are a component of the syndrome or are refractory to systemic treatment. The most common manifestation is periodontal disease, which occurs in up to 81% of patients. Other frequent oral manifestations include oral mucosal ulcers (14.3%), gingival/lip abscesses (4.8%), and periodontal abscesses (4.8%) [134].

#### 3.4.2. APECED

APECED (autoimmune polyendocrinopathy, candidiasis, and ectodermal dystrophy) is a rare autosomal recessive disorder caused by mutations in the AIRE gene, leading to autoimmunity and susceptibility to specific infections. It is characterized by the classical triad of hypoparathyroidism, adrenal insufficiency, and chronic mucocutaneous candidiasis. Infections such as chronic mucocutaneous candidiasis, COVID-19, bronchiectasis-associated pneumonia, and sepsis are significant clinical features of APECED, occurring alongside autoimmune symptoms. Hypoparathyroidism is the most prevalent (84.2%) autoimmune component and usually develops before 5 years of age. Chronic mucocutaneous candidiasis is typically the first manifestation of the APECED triad, usually presenting in childhood at a median age of 4 years. It is the second most prevalent of the triad and is present in 82.5% of patients with APECED [137,138,139].

### 3.5. Congenital Defects of Phagocyte Number or Function

According to the latest (2022) International Union of Immunological Societies (IUIS) classification of inborn errors of immunity (IEI), congenital defects of phagocyte number or function can be classified based on their major defect. Such categorization leads to the formation of groups such as those with congenital neutropenia (e.g., Elastase deficiency [severe congenital neutropenia 1], Kostmann Disease, or Shwachman–Diamond syndrome), those with defects in motility (e.g., Leukocyte adhesion deficiencies or Papillon–Lefèvre syndrome), and those with defects in respiratory burst (e.g., X-linked chronic granulomatous disease) along with other non-lymphoid defects [140].

A common hallmark of these inborn errors of immunity is an increased susceptibility to severe fungal (e.g., *Candida* and *Aspergillus*) and bacterial (e.g., *Staphylococcus aureus*, *Pseudomonas aeruginosa*, *Nocardia asteroides*, *Salmonella typhi*) infections. The most common sites of infection are the respiratory tract, lymph nodes, and skin (e.g., cellulitis or abscesses). Other manifestations include delayed separation of the umbilical cord, abnormal wound healing, and often, gingivitis or stomatitis. Furthermore, they can result in the development of autoimmune disorders or hematological malignancies. A significant proportion of patients present with growth failure [141,142]. The majority of primary phagocyte disorders are diagnosed in infancy due to the severity of the infection or the unusual presentation of the organism. However, some cases remain undiagnosed until adulthood [143].

It is crucial to emphasize the significance of dental hygiene practices, including regular dental check-ups and the incorporation of antibacterial mouthwashes such as chlorhexidine 0.2%, in preventing the onset of oral manifestations, such as gingivitis and periodontitis [141]. Patients are frequently treated with long-term antimicrobial prophylaxis, mainly trimethoprim–sulfamethoxazole (TMP–SMX). In the case of recurrent periodontitis, prophylactic antibiotics with activity against oral microflora should be administered, such as metronidazole, metronidazole with amoxicillin, or amoxicillin/clavulanic acid. In the case of fungal infections, itraconazole is preferred. Specifically for congenital neutropenias, granulocyte colony-stimulating factor (G-CSF) is recommended. For patients with chronic granulomatous disease (CGD) or defects of the IL-12/IFN-γ axis, interferon gamma (IFN-γ) is administered [141]. One of the most extensively researched treatment methods, particularly for CGD and leukocyte adhesion deficiency type I and III, is allogeneic hematopoietic stem cell transplantation (HSCT). However, this modality is associated with considerable adverse effects, including graft failure, graft-versus-host disease (GVHD), infections, and transplant-related mortality [144,145,146].

#### 3.5.1. Elastase Deficiency [Severe Congenital Neutropenia 1] and Kostmann Disease

Neutropenia is defined as a significant reduction in absolute neutrophil count (ANC) in the peripheral blood. Mild neutropenia is recognized when an absolute neutrophil count (ANC) ranges from 1.0 to 1.5 × 10^9^/L (1000–1500/µL). Moderate neutropenia falls within an ANC range of 0.5 to 1.0 × 10^9^/L (500–1000/µL), while severe congenital neutropenia (SCN) is defined by an ANC below 0.5 × 10^9^/L (<500/µL), which is also a manifestation in Kostmann Disease. The most prevalent causes of neutropenia are infection, immune disorders, and drugs. Congenital neutropenia, caused by germline mutations, is very rare, occurring in only about 10 million people worldwide. Moreover, not all mutations that trigger it have been identified. Severe congenital neutropenia affects approximately 1–2 cases per million individuals. The majority of cases are attributable to genetic defects, primarily linked to heterozygous mutations in ELANE (elastase deficiency [Severe congenital neutropenia 1]) and HAX1 deficiency (Kostmann Disease) [140,141]. This results in an increased apoptosis of myeloid progenitor cells in the bone marrow and/or neutrophils in the peripheral blood [141]. Individuals with SCN are at an increased risk of developing recurrent, often life-threatening infections, which typically emerge within the first few months of life. Among the most notable clinical features of SCN are abscesses, aphthous stomatitis, and gingivitis with gingival enlargement [141,147].

Given the protective function of neutrophils in periodontal tissues, patients with aggressive periodontitis often experience an early onset. This condition affects both primary and permanent dentition, with profound inflammation and substantial bone loss, often resulting in premature tooth loss [148]. For this reason, it is notable that despite the effective G-CSF therapy, periodontal symptoms may persist. Therefore, maintaining ongoing dental care is crucial. Once the integrity of the tooth–gum barrier, which prevents bacteria from penetrating below the enamel border, is compromised, it usually is unlikely to regenerate [141,149].

#### 3.5.2. Cyclic Neutropenia

Cyclic neutropenia (CyN), primarily induced by heterozygous mutations in the ELANE gene, is characterized by cyclic recurrences of severe neutropenia every 3 weeks and lasting only a few days, which may be due to a periodic change in the production of hematopoietic precursor cells by the bone marrow. In between these periods, patients typically present no symptoms and have ordinary physical examination results [141,148,150]. Prior to 2019. CyN was classified as a separate disease. However, the latest (2022) classification of inborn errors of immunity (IEI) by the International Union of Immunological Societies (IUIS) combines it with elastase deficiency (severe congenital neutropenia [SCN] 1) [140]. This distinction is particularly highlighted for the purposes of this scientific paper.

In dental clinics, CyN is frequently misdiagnosed as severe recurrent aphthous stomatitis or aggressive periodontitis due to similarities in their clinical presentation [151]. The mouth ulcers are deep and painful; hence, they are considered to be the most burdensome manifestation of CyN. Those ulcers often prevent patients from maintaining good oral hygiene. Ulcers are recurrent, deep, and self-limited, with a diameter greater than 10 mm and a depth usually greater than 4 mm. Subsequently, complications such as gingivitis and periodontitis frequently ensue [148,151]. Oral manifestations typically accompany painful cervical lymphadenopathy, sinusitis, otitis, pharyngitis, and bronchitis [150].

#### 3.5.3. Shwachman–Diamond Syndrome

Shwachman–Diamond syndrome (SDS) is a disorder caused by pathogenic variants (i.e., damaging mutations) in the SBDS gene or other genes that affect ribosome biogenesis and mitosis. It is a syndromic disorder that is characterized as a bone marrow failure syndrome (IBMFS) with manifestations such as exocrine pancreatic dysfunction, neutropenia, anemia or thrombocytopenia, sensorineural hearing loss, neurodevelopmental problems, and skeletal abnormalities [152,153].

The oral manifestations of Shwachman–Diamond syndrome have been thoroughly studied and found to result in a significantly higher prevalence of primary and permanent dental caries in individuals with SDS compared to healthy individuals. A review of the literature indicates that 65% of individuals diagnosed with SDS frequently experience oral ulcerations that recur. Within this group, 41% of individuals experienced them more than four times a year. Moreover, oral ulcers cause eating-related discomfort in 44% of affected individuals. Furthermore, nine out of 27 patients diagnosed with SDS have been identified as having delayed tooth development compared to zero cases in the 11-person control group [154]. This finding may be attributed by malabsorption, which is characteristic of SDS due to exocrine pancreatic insufficiency and discomfort during hygienic tasks due to pain caused by ulcerations [141,154].

#### 3.5.4. Leukocyte Adhesion Deficiency Type 1

This group of autosomal-recessive, primary immunodeficiencies is caused by abnormalities in the adhesion of leukocytes to endothelial cells. Such impairment impedes the migration of leukocytes from the bloodstream into infected or inflamed tissues. To date, three distinct leukocyte adhesion deficiency (LAD) types have been identified. All three types are characterized by neutrophilia, severe bacterial infections, and the absence of pus formation. LAD1 is the most prevalent form, with an incidence of one in a million individuals per year. In contrast, LAD2 and LAD3 have been documented in fewer than 30 cases worldwide [141,148,155].

LAD1 is caused by mutations in the gene encoding CD18, the β chain of leukocyte β2 integrins, resulting in impaired migration and chemotaxis of all leukocytes. The oral manifestations of this disease result rather from dysregulated overexpression of the cytokine IL-17 than from defective neutrophil surveillance itself, suggesting a role for neutrophils in regulating the immune response and opening new therapeutic options. This mechanism is associated with the disruption of a major neutrophil homeostatic mechanism known as “neutrostat”, which aims to attract neutrophils into the tissue through IL-23/IL-17, triggering neutrophil recruitment by inducing G-CSF and neutrophil chemoattractants. If successful, apoptosis of tissue neutrophils and engulfment by tissue macrophages become the signal for the downregulation of the IL-23/IL-17 axis, operating via a negative feedback mechanism. In LAD1, where neutrophils cannot migrate to peripheral tissues, this regulatory mechanism is disrupted, leading to excessive secretion of IL-23, IL-17, and G-CSF. Their overproduction explains the increased granulopoiesis and blood neutrophilia in LAD1 patients, resulting in inflammatory periodontal bone loss as well as periodontitis. Moreover, IL-17-dependent inflammation promotes bacterial overgrowth in this condition, suggesting microbial triggering of inflammatory responses [147,156,157]. Recently, the administration of an anti-IL12/IL23 monoclonal antibody—ustekinumab—led to a notable decrease in oral inflammation with no significant adverse effects and a significant improvement in non-healing cutaneous ulcers. This proved the role of interleukin-23-dependent production of interleukin-17 in LAD1, opening new therapeutic avenues that may save patients with LAD1 from premature loss of primary or permanent teeth [156,158].

#### 3.5.5. Papillon–Lefèvre Syndrome

Papillon–Lefèvre syndrome (PLS) is defined by the occurrence of early-onset periodontitis and palmoplantar hyperkeratosis. PLS is caused by mutations in the CTSC gene, which encodes Cathepsin C, a lysosomal enzyme involved in epidermal differentiation and desquamation, as well as in the activation of serine proteases found in phagocytes. This diminished chemotactic and phagocytic capacity of neutrophils impairs the gingiva’s local defense mechanisms against oral bacteria resulting in severe gingivitis, chronic periodontal inflammation, and subsequent tooth loss [141]. On the contrary to most IEI, only 15–20% of patients are predisposed to recurrent systemic infections [159].

Patients with PLS present the first clinical signs in the oral cavity as soon as the deciduous teeth erupt. Periodontal manifestations, such as severe periodontitis, plaque accumulation, caries, generalized bone loss, and tooth mobility, become apparent by the age of 2 to 3 years. The gingival tissues surrounding the deciduous teeth exhibit inflammation, swelling, tenderness upon touch, and the presence of periodontal abscesses. Typically, patients lose their primary teeth prematurely by the age of 5. Subsequent to the loss of primary teeth, gingival inflammation resolves, and the oral mucosa appears healthy until the eruption of succedaneous teeth [147,160].

Subsequently, severe gingival inflammation with elevated bleeding indexes ensues, accompanied by plaque accumulation, which culminates in the formation of deep periodontal pockets. Dental elements exhibit high mobility, anterior group fluttering, and loss of vertical dimension. Spontaneous loss of dental elements is a common occurrence. Panoramic radiographs demonstrate advanced bone loss, which gives the teeth a characteristic “floating in air” appearance. The patients exhibited a reduction in facial height, a senile appearance, lymphadenopathy, and halitosis [160,161].

Treatment of PLS patients is symptomatic and usually ineffective. It includes eliminating the reservoir of bacteria, using conventional periodontal treatment, e.g., scaling, at-home oral hygiene, antiseptic mouth rinses, and systemic antibiotic therapy. During the growth period, intensive orthodontic and prosthetic therapy is necessary, and patients eventually require complete mobile prostheses on both arches. Resistance to antibiotic treatment presents a notable challenge in managing PLS [160,162].

#### 3.5.6. X-Linked Chronic Granulomatous Disease

The etiology of X-linked chronic granulomatous disease (CGD) is a defective function of the NADPH oxidase enzyme complex which arrests the initiation of phagocyte respiratory burst pathway and generation of reactive oxygen species (ROS), which are taking part in microbial destruction. Given that approximately two thirds of genetic defects of the NADPH oxidase complex are X-chromosome-linked, men are considerably more prone to CGD, accounting for approximately 80% of patients with CGD [141,163].

Among the symptoms that may be observed are recurrent, severe bacterial and fungal infections, inflammatory complications such as granuloma formation, colitis, and other typical congenital defects of phagocyte number or function; oral manifestations also occur. These oral manifestations encompass recurrent aphthous ulcerations, periodontitis, gingivitis, and gingival hypertrophy. Patients with CGD are also more susceptible to oral fungal infections [106]. Factors that predispose those individuals to oral complications include neutrophil dysfunction, immunosuppressive therapies, and malnutrition due to gastrointestinal symptoms [148,164].

### 3.6. Defects in Intrinsic and Innate Immunity

This group of primary immunodeficiencies includes a range of syndromes associated with pattern-recognizing receptors (PRRs). These are genetic diseases that are inherited autosomally, recessively or predominantly. Such defects may encompass various aspects of immunity, such as immune response to infections, regulation of immune response, and ability to respond to pathogens [140,165].

#### 3.6.1. Mendelian Susceptibility to Mycobacterial Disease (MSMD)

Mendelian susceptibility to mycobacterial disease (MSMD) is a rare condition where individuals exhibit a predisposition to clinical disease caused by weakly virulent mycobacteria, despite normal hematological and immunological test results. MSMD is caused by inborn errors of IFN-γ immunity. Patients with MSMD demonstrate significant genetic heterogeneity. Mutations in 15 different genes (IFNGR1, IFNGR2, STAT1, JAK1, IRF8, SPPL2A, IL12B, IL12RB1, IL12RB2, IL23R, ISG15, TYK2, RORC, CYBB, and NEMO) have been identified as the cause of isolated or syndromic MSMD. The mutations define 30 disorders with high locus and genetic allelic heterogeneity, with striking physiological homogeneity. In almost half of all MSMD patients, a genetic etiological factor remains unidentified. Besides susceptibility to mycobacterial infections, MSMD patients may also experience salmonellosis, candidiasis, tuberculosis, and occasionally, infections from other intramacrophagic bacteria, fungi, parasites, and potentially, viruses. Additionally, patients may experience selective susceptibility and disease symptoms resulting from exposure to attenuated *Mycobacterium bovis* Bacille Calmette–Guérin (BCG) vaccines. Some patients may derive benefit from IFN-γ therapy in addition to antibiotics. Oral manifestations include chronic mucocutaneous candidiasis and recurrent oropharyngeal candidiasis [81,140,166].

#### 3.6.2. Epidermodysplasia Verruciformis (EV)

Epidermodysplasia verruciformis (EV) is a rare genodermatosis that increases the risk of cutaneous malignancies resulting from infections. Mutations in TMC6 or TMC8 genes render individuals susceptible to human papillomavirus (HPV) infections and the characteristic plane warts seen in EV. Individuals affected by this condition, particularly those who are exposed to UV radiation, have a lifelong elevated risk of developing cutaneous malignancies, with squamous cell carcinoma (SCC) being a notable example. To the best of our knowledge, there have been no reported cases of HPV-related EV lesions or cancers occurring in the oral cavity, though they may manifest on the facial skin [140,167].

#### 3.6.3. Predisposition to Severe Viral Infection

Predisposition to severe viral infection refers to an individual’s increased susceptibility to developing severe or life-threatening symptoms following exposure to viral pathogens. This predisposition can arise from various factors such as genetic mutations affecting the immune system, underlying health conditions, immunosuppression, or age-related vulnerabilities. Viral infections, especially those caused by herpes viruses, may appear in the oral cavity. However, due to the severity of infections seen in patients with pathogenic variants in the IFN signaling pathway, oral involvement is rarely documented. Although HSV-1 gingivostomatitis has been observed in STAT2 deficient patients, specific oral characteristics have not been reported in patients with deficiencies in IFNAR1/2, IRF7/9, MDA5, or RNA polymerase III [168,169].

#### 3.6.4. Herpes Simplex Encephalitis (HSE)

Herpes simplex encephalitis (HSE) is a rare but serious viral infection of the brain caused by the herpes simplex virus (HSV), primarily HSV-1. It is considered a medical emergency due to its potential to cause severe neurological damage and death if not promptly diagnosed and treated. HSE typically presents with symptoms such as fever, headache, altered mental status, seizures, and focal neurological deficits. Interestingly, children with herpes simplex encephalitis (HSE) do not demonstrate heightened susceptibility to HSV-1-related conditions affecting areas other than the central nervous system (CNS), such as herpes gingivostomatitis, which is the most prevalent clinical manifestation of HSV-1 infection in the general population [168,169,170].

#### 3.6.5. Predisposition to Invasive Fungal Diseases

Predisposition to invasive fungal diseases refers to an individual’s increased susceptibility to developing severe fungal infections that penetrate and spread within the body’s tissues or organs. Oral candidiasis, which is a component of chronic mucocutaneous candidiasis (CMC) caused by *Candida* albicans, is often linked with CARD9 deficiency and can serve as an indicator of the disease. Recent reviews report its occurrence in nearly 30% of patients. Prophylactic use of oral fluconazole can help prevent CMC, and regular thorough screening of the oral mucosa is essential for detecting its onset or recurrence [140,171].

#### 3.6.6. Predisposition to Mucocutaneous Candidiasis (CMC)

Chronic mucocutaneous candidiasis (CMC) manifests as recurrent or persistent infections of the mucous membranes and skin caused by *Candida* fungi, while hypothyroidism typically arises from autoimmune thyroiditis. Individuals with these conditions are prone to developing additional infectious diseases, such as viral, bacterial, and fungal infections, as well as other autoimmune disorders, such as enterocolitis, immune cytopenia, endocrinopathies, and systemic lupus erythematosus. Due to disturbances in the IL-17 axis, individuals with chronic mucocutaneous candidiasis (CMCD) exhibit a high susceptibility to *Candida* albicans mucosal infections, with nearly 100% experiencing recurrent or severe candidiasis of the oral mucosa, including conditions like thrush, glossitis, and cheilitis. Therefore, it is recommended to investigate germline mutations affecting the IL-17 signaling pathway in patients with recurrent oral candidiasis. Additionally, a third of patients with STAT1 gain-of-function (GOF) pathogenic variants may also suffer from mucocutaneous viral infections, such as herpes simplex virus 1 (HSV-1) gingivostomatitis. Treatment typically involves appropriate systemic or topical antifungal and systemic antiviral agents. Moreover, patients with CMCD, especially those with autosomal dominant (AD) STAT1 GOF pathogenic variants, have an elevated risk of oral and esophageal squamous cell carcinoma, partly due to chronic inflammation associated with persistent CMC, necessitating regular oral mucosa examinations. Oral anomalies, including progressive macroglossia, macrocheilitis, and dental abnormalities such as peg-shaped incisors, have been observed in ACT1 deficiency. It has been reported that rare instances of delayed exfoliation of primary molars and enamel erosions have been observed in individuals with a STAT1 GOF mutation. However, further investigation is necessary to determine the relationship between STAT1 germline mutations and these tooth anomalies that are commonly observed in the general population. The delayed exfoliation of primary teeth and dental crowding observed in families with JNK1 haploinsufficiency may be attributed to impaired TGFβ-dependent homeostasis of connective tissues [140,171,172].

#### 3.6.7. TLR Signaling Pathway Deficiency with Bacterial Susceptibility

Toll-like receptors (TLRs) play crucial roles in initiating the immune response by recognizing microbial or host-derived molecules, particularly in patients with primary immune deficiencies (PIDs) affecting TLR signaling. Defects in specific TLR signaling components like interleukin-1 receptor-associated kinase-4 and myeloid differentiation factor 88 (MyD88) increase susceptibility to bacterial infections, while mutations in nuclear factor-κB essential modulator (NEMO) and downstream mediators broaden susceptibility to bacteria, viruses, and fungi. Additionally, alterations in TLR signaling pathways, such as those seen in common variable immunodeficiency and chronic granulomatous disease, illustrate the complex interplay between TLR responses and immune system dysregulation in various PID conditions [140,171].

### 3.7. Autoinflammatory Disorders

Systemic autoinflammatory disorders, a rare and expanding group of conditions, result from innate immune system dysfunction, characterized by recurrent fever attacks, cutaneous signs, chest or abdominal pain, lymphadenopathy, vasculopathy, and musculoskeletal symptoms. While typically diagnosed in childhood, an increasing number of adult patients, including those with adult-onset disease, are being identified with these disorders [140,141,142,143,144,145,146,147,148,149,150,151,152,153,154,155,156,157,158,159,160,161,162,163,164,165,166,167,168,169,170,171,172,173].

#### 3.7.1. PFAPA Syndrome

The syndrome of periodic fever, aphthous stomatitis, pharyngitis, and cervical adenitis (PFAPA syndrome) is the most common cause of periodic fever in childhood, typically presenting before the age of 5 and characterized by episodes of high fever lasting 3–7 days that recur every 2–8 weeks, accompanied by aphthous stomatitis, pharyngitis, and/or cervical adenitis. The disease generally resolves by adolescence, with patients being asymptomatic and showing regular growth between episodes. The disease itself is caused by cytokine dysfunction and potentially has a genetic basis. It is not uncommon for patients to be treated with antibiotics without any discernible effect. In contrast, improvement is observed even after a single oral dose of corticosteroids [174,175].

#### 3.7.2. Familial Mediterranean Fever (FMF)

Familial Mediterranean fever (FMF) is the most common hereditary autoinflammatory disease, predominantly affecting populations from the Eastern Mediterranean but reported worldwide. FMF is caused by gain-of-function mutations in the MEFV gene, which encodes the pyrin protein, FMF is characterized by recurrent, short episodes of fever, peritonitis, pleuritis, arthritis, and rash, with oral manifestations occurring in up to 10% of cases. Although the condition is self-limiting, it can lead to severe long-term complications. Oral ulcers are frequent in patients with this condition. The prevalence of moderate to severe periodontitis is higher in cases of familial Mediterranean fever in patients with amyloidosis than in patients without amyloidosis. Periodontitis may serve as a significant, yet latent, source of chronic inflammation, which in turn elevates the levels of acute phase reactants in patients with familial Mediterranean fever. This may also have an impact on the development of amyloidosis in patients with FMF. Treatment of periodontitis may be beneficial in alleviating the disease burden in patients with familial Mediterranean fever [176,177,178]. A significant correlation between homozygous mutation of the M694V gene and periodontitis in patients with FMF has been established (*p* < 0.05). These findings suggest that the presence of a homozygous M694V gene mutation may increase the risk of periodontitis in patients with FMF [179].

#### 3.7.3. Mevalonate Kinase Deficiency (MKD)

Hyperimmunoglobulinemia D and periodic fever syndrome (HIDS), now known as mevalonate kinase deficiency (MKD), is an autoinflammatory disease characterized by recurrent episodes of fever, cervical lymphadenopathy, hepatomegaly, splenomegaly, abdominal pain, skin rash, and arthralgia. These attacks are accompanied by increased inflammatory markers like C-reactive protein (CRP) and serum amyloid A (SAA) and can be triggered by childhood vaccinations or minor infections, though the specific triggers remain largely unknown. The term “HIDS” was derived from elevated immunoglobulin D (IgD) levels observed in early patients. However, the term “MKD” is now more accurate, given the established genetic basis. In patients with this condition, oral ulcers are a common occurrence [106,180,181].

### 3.8. Complement Deficiencies

The root cause of complement system component deficiencies are gene mutations inherited predominantly autosomally recessively, and much less frequently, autosomally dominantly, and in one case, passed on via the X chromosome. A genetic defect can cause a deficiency, usually partial, of any component of the complement system. The frequency of deficiencies of individual components is unknown. However, primary immunodeficiencies of this group are classified as rare diseases. The current IUIS classification includes 30 primary immunodeficiencies of this type. In clinical practice, deficiencies of the C3c and C4 components are the most commonly encountered. The clinical manifestations of these deficiencies are associated with the absence of specific components of the complement system. Deficiencies of the so-called early complement fractions (i.e., C1q, C1r, C1s, C2, C3, C4) increase the risk of developing autoimmune diseases (e.g., systemic lupus erythematosus, glomerulonephritis). At the same time, C2 and C3 have been linked to an elevated risk of infection with enveloped bacteria. Defects in C5–C9 components, properdin, or factor D are associated with increased susceptibility to meningococcal infections. Deficiencies in regulatory factors (factors H and I) can cause glomerulonephritis. Patients with C1 esterase inhibitor deficiency are not immunocompromised but have hereditary angioedema (HAE), which can be life-threatening, especially with laryngeal edema [140,182,183].

The evidence linking the complement system to periodontitis is indisputable. Clinical and histological observations indicate a correlation between periodontal inflammatory activity and local complement activation. Consequently, certain gene polymorphisms or deficiencies of specific complement components predispose to increased susceptibility to periodontitis. Partial C4 gene deficiencies were significantly more frequent in periodontal patients compared to healthy controls, analogous to a C5 single nucleotide polymorphism (rs17611) [184,185,186]. A case of angioedema resulting from C1-esterase inhibitor deficiency localized only to the free gingiva has also been described [187].

### 3.9. Bone Marrow Failure

Bone marrow failure (BMF) syndromes represent a diverse range of non-malignant hematologic disorders characterized by decreased blood cell production, which can be attributed to both inherited and acquired factors. Immune dysregulation, including aberrant T or B cell responses, is implicated in BMF pathophysiology, with hematological improvement following standard immunosuppressive or anti-complement therapies serving as indirect evidence of the immune system’s central role [106,140].

An example is Fanconi anemia, which is an autosomal recessively inherited disease characterized by progressive pancytopenia and congenital skeletal malformations. Patients have an increased propensity for caries. There may be aggressive periodontitis, cases of extra teeth, or congenital absence of teeth have been described. The increased susceptibility to periodontal disease in patients with Fanconi anemia may be due not only to the characteristics of anemia, leukopenia, and abnormal oxygen radical detoxification characteristic of the disease itself but also to the use of drugs used during intensive immunosuppressive treatment, such as prednisolone [188,189].

### 3.10. Phenocopies of Inborn Errors of Immunity

This is a group of diseases whose clinical manifestations mimic IEIs caused by known genetic defects but do not show their presence. They may be due to the action of autoantibodies that inhibit the functions of specific proteins (i.e., cytokines, receptors, signaling proteins) or to a somatic mutation originating in the postzygotic period (associated with somatic mosaicism) identical to the genetic error that determines the IEIs [140].

Periodontal lesions may occur in chronic mucocutaneous candidiasis (CMC) or Good syndrome. In CMC, where autoantibodies against IL-17 and/or IL-22 are present, long-standing multiple mucosal and tongue fungal infections may occur [43]. Good syndrome is defined by the presence of a thymoma, hypogammaglobulinemia, low number of peripheral B cells, and variably, peripheral CD4 T cell lymphopenia and inverted CD4/CD8 T cell ratio, associated with infections and autoimmune diseases. Interestingly, periodontitis and oral erosive lichen planus may also be present in this syndrome [190,191].

## 4. Diagnosing IEIs

In diagnosing IEIs, of paramount importance is the patient’s history and consideration of deficiency occurrence possibility. Years of clinical immunology practice indicate that diagnoses of IEIs are established by physicians who have encountered patients with IEIs in the past and/or have otherwise sought to expand their knowledge about IEIs. Fortunately, there has been a gradual increase in awareness among physicians of IEIs, as evidenced by the fact that the majority of IEIs have been diagnosed within the last decade (in the study conducted by the author—up to 70%). A comprehensive diagnostic scheme for IEIs, for reference by physicians who are not clinical immunologists, is provided in Table 4.

## 5. Prophylaxis and Management of IEIs

In all cases of gingivitis, periodontal treatment involves professional removal of dental plaque. In healthy individuals, plaque removal should be performed at least 1–2 times a year. In patients with IEIs with concomitant periodontal disease, these check-ups should take place more often, at least 3–4 times a year, although, of course, each case should be considered individually. It is also necessary to place special emphasis on educating patients about daily oral care.

Therapeutic management is multidirectional and involves many aspects, not solely medical. A balanced diet in accordance with the principles of healthy nutrition, regular physical activity adapted to age, condition, and health status, performing regular check-ups and consultations, vitamin D3 substitution, and avoiding exposure to human concentrations should be included. It is contraindicated for patients to travel to locations with an elevated sanitary–epidemiological risk. It is inadvisable to delay the inclusion of an antibiotic with prolonged infection. In the event of the necessity for transfusion of blood products, transfuse exposed, leukocyte-poor preparations [192].

It is highly pertinent to implement supplementary vaccination due to its pleiotropic effect, not only in the immunodeficient individuals themselves but also in their immediate environment (cocoon vaccination). Live vaccines are generally not recommended in patients with cellular immunity disorders and severe humoral immune disorders (i.e., XLA, CVID), although each case should be considered individually. There are no contraindications to their use in mild antibody deficiencies, i.e., selective IgA deficiency, IgG subclass/y deficiency, or specific antibody deficiency. Killed (inactivated) vaccines, such as influenza virus vaccination, are recommended, as are protein and polysaccharide vaccines (against S. pneumoniae, H. influenzae, meningococcus, and tetanus) [193].

In some cases, patients may require the administration of immunoglobulin preparations. The indications for their use include deficiencies in IgG concentrations, specific antibody deficiency (SPAD), and, in some cases, the presence of acute autoimmune diseases. The extent of the reduction in IgG concentration is not the sole determining factor in the decision to initiate treatment, and instead, it is the patient’s clinical condition that serves as the primary indicator [194]. It should be borne in mind that immunoglobulins are prepared from a pool of plasma from at least 1000 donors. In recipients of such preparations, IgG class antibodies (e.g., anti-HCV, ANA) are determined by immunoglobulin donor antibodies. A reliable diagnosis can only be made 4–6 weeks after the withdrawal of the preparation, provided that antibody production in this class is not disrupted.

It is important to consider the necessity of chronic drug prophylaxis in some patients. Infectious complications associated with IEIs that necessitate continuous antimicrobial prophylaxis include chronic granulomatous disease, hyper-IgE syndrome, and some IEIs that manifest with neutropenia and/or severe chronic/recurrent infections and/or specific complications, such as bronchial dilatation. Some patients with IEIs may require prophylactic antibiotic therapy on a periodic basis, for example, during the fall and winter months, following tooth extractions, during ENT procedures, and in the event of other procedures involving tissue disruption. In some cases, patients with IEI may require prolonged antibiotic therapy for the treatment of specific infections, such as sinusitis, otitis media, and pneumonia. Microbe-dependent prophylaxis is indicated for the following microorganisms: staphylococci (typically trimethoprim–sulfamethoxazole), streptococci, mycoplasma, and nontuberculous mycobacteria (usually azithromycin). Patients with IEIs who require continuous antifungal prophylaxis include those with chronic granulomatous disease, hyper-IgE syndrome, or chronic cutaneous-mucosal candidiasis. The specific antifungal treatment regimen is also contingent upon the identified microorganism: *Aspergillus* infections are typically treated with itraconazole, while *Candida* infections are usually treated with fluconazole. In some cases, patients may require prolonged treatment periods, such as those associated with fungal pneumonia or central nervous system (CNS) mycosis. Deficiencies that require continuous antiviral prophylaxis include SCID and DOCK8 deficiency. Some patients require prolonged therapy, typically spanning several months, to prevent infections caused by herpes simplex virus, VZV, or CMV. Acyclovir is the antiviral medication of choice for HSV and VZV infections, whereas ganciclovir is the preferred agent for CMV infections [195,196,197,198].

Naturally, treatment in this group of patients often does not differ from that used in immunocompetent patients. This encompasses the utilization of both local and systemic pharmacological agents. Nevertheless, the administration of appropriate treatment, including the expeditious implementation of therapy, is crucial in patients with IEI. It prevents complications and the spread of lesions due to the fact that the oral cavity, in addition to the nasal cavity and skin, is the most common portal of infection.

Very important for dentists, although they do not occur frequently, are IEIs that may result in tooth loss [199]. For this reason, they are included in a separate table along with the recommended treatment options (Table 5). It should be remembered that patients with these conditions should be treated by periodontologists.

## 6. Conclusions

Clinical immunology is a rapidly growing field of medicine. Currently, nearly 500 disease entities are classified as IEI, and the list is expanding annually. Much remains to be done in the area of the relationship between oral diseases and IEIs, including further research into the mechanisms responsible for their occurrence. Unfortunately, there is little data on this subject in the literature. Currently, it is most often assumed that changes in the oral cavity are the result of a disorder of a specific type of immunity (e.g., cellular or humoral immunity) occurring in a given disease or the result of the treatment used. However, this is a far too simplified assumption. A proper diagnosis, but more importantly, an early diagnosis, determines the quality and length of life of patients with IEI. Unfortunately, the estimated delay from the onset of first symptoms to the diagnosis of IEI is 16.1 years [208]. In the case of the most prevalent group of IEIs, primary antibody deficiencies, the estimated delay from the onset of the initial symptoms to a diagnosis is 6–12 years [209]. The key to recognizing IEI is to consider the possibility of its occurrence.

## Figures and Tables

**Table 1:** C–lassification of the various IEIs and their subtypes (Adapted [28]). SCID—Severe Combined Immunodeficiency; CID—Combined Immunodeficiency; HIES—Hyper IgE Syndromes; EDA-ID—Anhidrotic Ectodermodysplasia with Immunodeficiency; CVID—Common Variable Immunodeficiency; FHL—Familial Hemophagocytic Lymphohistiocytosis; ALPS—Autoimmune Lymphoproliferative Syndrome; EBV—Epstein–Barr Virus; MSMD—Mendelian Susceptibility to Mycobacterial Disease; HPV—Human Papillomavirus; HSE—Herpes Simplex Encephalitis; TLR—Toll-like Receptor.

Inborn Error of Immunity	Subtypes
Immunodeficiencies affecting cellular and humoral immunity	1. T-B+ SCID 2. T-B-SCID 3. CID, Generally Less Profound than SCID
Combined immunodeficiencies with associated or syndromic features	Immunodeficiency with Congenital Thrombocytopenia DNA Repair Defects Thymic Defects with Additional Congenital Anomalies Immuno-osseous Dysplasias Hyper IgE Syndromes (HIES) Defects of Vitamin B12 and Folate Metabolism Anhidrotic Ectodermodysplasia with Immunodeficiency (EDA-ID) Calcium Channel Defects Other Defects
Predominantly antibody deficiencies	Severe Reduction in All Serum Immunoglobulin Isotypes with Profoundly Decreased or Absent B Cells, Agammaglobulinemia Severe Reduction in at Least 2 Serum Immunoglobulin Isotypes with Normal or Low Number of B Cells, CVID Phenotype Severe Reduction in Serum IgG and IgA with Normal/Elevated IgM and Normal Numbers of B cells, Hyper IgM Isotype, Light Chain, or Functional Deficiencies with Generally Normal Numbers of B Cells
Diseases of immune dysregulation	Familial Hemophagocytic Lymphohistiocytosis (FHL syndromes) FHL Syndromes with Hypopigmentation Regulatory T Cell Defects Autoimmunity with or without Lymphoproliferation Immune Dysregulation with Colitis Autoimmune Lymphoproliferative Syndrome (ALPS, Canale-Smith syndrome) Susceptibility to EBV and Lymphoproliferative Conditions
Congenital defects of phagocyte number or function	Congenital Neutropenias Defects of Motility Defects of Respiratory Burst Other Non-Lymphoid Defects
Defects in intrinsic and innate immunity	Mendelian Susceptibility to mycobacterial disease (MSMD) Epidermodysplasia verruciformis (HPV) Predisposition to Severe Viral Infection Herpes Simplex Encephalitis (HSE) Predisposition to Invasive Fungal Diseases Predisposition to Mucocutaneous Candidiasis TLR Signaling Pathway Deficiency with Bacterial Susceptibility Other Inborn Errors of Immunity Related to Non-Hematopoietic Tissues Other Inborn Errors of Immunity Related to Leukocytes
Autoinflammatory disorders	Type 1 Interferonopathies Defects Affecting the Inflammasome Non-Inflammasome Related Conditions
Complement deficiencies	Deficiencies of individual components of the complement system
Bone marrow failure	Fanconi Anemia Dyskeratosis Congenita MECOM deficiency
Phenocopies of inborn errors of immunity	VEXAS (vacuoles, E1 enzyme, X-linked autoinflammatory, somatic) syndrome Chronic mucocutaneous candidiasis Severe COVID-19

**Table 2:** Symptoms that may indicate inborn errors of immunity—according to the Jeffrey Modell Foundation [29].

Symptoms That May Indicate Inborn Errors of Immunity in Children	Symptoms That May Indicate Inborn Errors of Immunity in Adults
1. Four or more new ear infections within 1 year.	1. Two or more new ear infections within 1 year.
2. Two or more serious sinus infections within 1 year.	2. Two or more new sinus infections within 1 year, in the absence of allergy.
3. Two or more months on antibiotics with little effect.	3. One pneumonia per year for more than 1 year.
4. Two or more pneumonias within 1 year.	4. Chronic diarrhea with weight loss.
5. Failure of an infant to gain weight or grow normally.	5. Recurrent viral infections (colds, herpes, warts, condyloma).
6. Recurrent, deep skin or organ abscesses.	6. Recurrent need for intravenous antibiotics to clear infections.
7. Persistent thrush in mouth or fungal infection on skin.	7. Recurrent, deep abscesses of the skin or internal organs.
8. Need for intravenous antibiotics to clear infections.	8. Persistent thrush or fungal infection on skin or elsewhere.
9. Two or more deep-seated infections including septicemia.	9. Infection with normally harmless tuberculosis-like bacteria.
10. A family history of primary immunodeficiency.	10. A family history of primary immunodeficiency.

**Table 3:** Inborn errors of immunity associated with periodontal and dental diseases. XL—X-linked inheritance; AR—autosomal recessive inheritance; AD—autosomal dominant inheritance; GOF gain-of-function; LOF—loss-of-function; CD40—Cluster of Differentiation 40; CD154—Cluster of Differentiation 154; HIGM—Hyper-IgM Syndrome; IgM—Immunoglobulin M; MHC—Major Histocompatibility Complex; SCID—Severe Combined Immunodeficiency; SASH3—SAM and SH3 Domain Containing 3; SAM—Sterile Alpha Motif; SH3—SRC Homology 3 Domain; AD-HIES—Autosomal Dominant Hyper-Ige Syndrome; STAT3—Signal transducer and activator of transcription 3; AT—Ataxia-Telangiectasia; DGS—DiGeorge Syndrome; EDA-ID—Anhidrotic Ectodermal Dysplasia with Immune Deficiency; NEMO—Necrotic Factor Kappa B Essential Modulator; IKBKG—Inhibitor of Nuclear Factor Kappa B Kinase Regulatory Subunit Gamma; IKBA—Nuclear Factor of Kappa Light Polypeptide Gene Enhancer in B-cells Inhibitor Alpha; GOF—Gain of Function; EPG5—Ectopic P-Granules 5 Autophagy Tethering Factor; IL6ST—Interleukin 6 Signal Trandsucer; TGFBR—Transforming Growth Factor Beta Receptor; WAS—Wiskott–Aldrich Syndrome; ZNF341—Zinc Finger Protein 341; AR-HIES—Autosomal Recessive Hyper-Ige Syndrome; CVD—Common Variable Immunodeficiency; IgG—Immunoglobulin G; IgG3—Immunoglobulin G 3; SIgAD—Selective IgA Deficiency; IgA—Immunoglobulin A; THI—Transient Hypogammaglobulinemia of Infancy; XLA—X-Linked Agammaglobulinemia; APECED—Autoimmune Polyendocrinopathy–Candidiasis–Ectodermal Dystrophy; APS-1—Autoimmune Polyendocrine Syndrome 1; GS2—Griscelli Syndrome type 2; CGD—Chronic Granulomatous Disease; GA-TA2—GATA-Binding Factor 2; GATA—Guanine–Adenine–Thymine–Adenine; HPV—Human Papilloma Virus; JAGN1—Jagunal Homolog 1; SCN6—Sodium Channel Protein Type 6; LAD1-3—Leukocyte Adhesion Deficiency Type 1–3; SCNs—Severe Congenital Neutropenias; SDS—Shwachman–Diamond Syndrome; SMARCD2—SWI/SNF-related Matrix-Associated Actin-dependent Regulator of Chromatin subfamily D Member 2; SWI/SNF—Switch/Sucrose Non-Fermentable; SGD2—Specific Granule Deficiency 2; VPS45—Vacuolar Protein Sorting 45; SCN5—Sodium Channel Protein Type 5; WDR1—WD Repeat Domain 1; WD—Tryptophan–Aspartic Acid; CMC—Chronic Mucocutaneous Candidiasis; HSV-1—Herpes Simplex Virus 1; CARD9—Caspase Recruitment Domain-Containing Protein 9; CD16—Cluster of Differentiation 16; IFN—Interferon; IRAK-4—Interleukin-1 Receptor-Associated Kinase 4; MyD88—Myeloid Differentiation Primary Response 88; MSMD—Mendelian Susceptibility to Mycobacterial Disease; WHIM—Warts, Hypogammaglobulinemia, Immunodeficiency, Myelokathexis; CANDLE—Chronic Atypical Neutrophilic Dermatosis with Lipodystrophy and Elevated temperature; FMF—Familial Mediterranean Fever; MKD—Mevalonate Kinase Deficiency; IgD—Immunoglobulin G; PFAPA—Periodic Fever, Aphthous stomatitis, Pharyngitis and Adenitis; SAMHD1—SAM and HD Domain Containing Deoxynucleoside Triphosphate Triphosphohydrolase 1; HD—Histidine- Aspartic; C1—Complement Component 1; C3—Complement Component 3; C4—Complement Component 4; C5—Complement Component 5.

Disease	Associated Pathology	Typical Age (Lifetime) of Onset	Inheritance
**IMMUNODEFICIENCIES AFFECTING CELLULAR AND HUMORAL IMMUNITY**			
CD40 ligand (CD154) deficiency and CD40 deficiency (formerly Hyper-IgM syndrome—HIGM) [30]	ulcers can begin to erode the gingiva and may mimic periodontal disease, increased risk of infection.	One year old in 50% of males, 90% to four years old	XL AR
MHC (Class I and II) deficiency [31,32]	oral herpes.	MHC class I deficiency: Childhood MHC class II deficiency: Infancy, neonatal	AR
SASH3 deficiency [33,34]	mucosal infections.	Childhood	XL
Severe combined immunodeficiency (SCID) [35]	candidiasis, herpes infections, oral ulcerations, severe necrotizing gingivostomatitis.	Infancy, neonatal	AR/XL/AD/GOF
**COMBINED IMMUNODEFICIENCIES WITH ASSOCIATED OR SYNDROMIC FEATURES**			
AD-HIES STAT3 deficiency (Job syndrome) [36]	ligneous gingivitis/periodontitis, chronic mucocutaneous candidiasis, retained primary teeth, “double rows” of teeth due to primary teeth failure to exfoliate on the eruption of the permanent dentition, angular cheilitis, recurrent aphthous ulceration, generalized aggressive periodontitis, arched palate.	Infancy, neonatal	AD
Ataxia–telangiectasia (A–T) [37]	periodontal disease, Herpetic gingivostomatitis, candidiasis.	Childhood, infancy	AR
DiGeorge syndrome (DGS) [38]	delayed formation and eruption of permanent teeth, aberrant tooth shape, and enamel hypoplasia along with enamel hypocalcification, dental caries with multiple active incipient caries lesions, altered eruption patterns.	All ages	AD
EDA-ID due to NEMO/IKBKG deficiency (ectodermal dysplasia, immune deficiency) [39]	variable defects of teeth, anodontia/hypodontia, delayed eruption of teeth and conical incisors.	Infancy, neonatal	XL
EDA-ID due to IKBA GOF mutation [39]	variable defects of teeth.	Infancy, neonatal	AD
EPG5 deficiency (Vici syndrome) [40]	chronic mucocutaneous candidiasis.	Antenatal, neonatal	AR
IL6 signal transducer (IL6ST) deficiency (partial) [41,42]	retention of primary teeth.	Childhood	AR
IL6ST deficiency (complete) [41,43]	tooth retention due to connective tissue defects.	Childhood	AR
Loeys–Dietz syndrome (TGFBR deficiency) [44]	retention of primary teeth.	Infancy, neonatal	AD
Schimke’s immuno-osseous dysplasia (SIOD) [45]	microdontia, hypodontia, short and thin tooth roots, malformed primary and/or permanent molars.	Antenatal, neonatal, infancy	AR
Wiskott–Aldrich syndrome (WAS) [46]	gingivitis, periodontitis, petechiae in oral mucosa, gingival ulceration, bleeding in the oral cavity.	Infancy, neonatal	XL
ZNF341 deficiency AR-HIES [47,48]	retention of primary teeth.	Occurs at a younger age than AD-HIES	AR
**PREDOMINANTLY ANTIBODY DEFICIENCIES**			
Common variable immunodeficiency (CVID) [49]	necrotizing ulcerative periodontitis, aphthae, lichenoid lesions, gingivitis, ↑ odontogenic infection, possible septicemia from odontogenic infection.	All ages	Variable
Isolated IgG subclass deficiency [50]	↑ susceptibility to viral infections in IgG3 deficiency.	All ages	Unknown
Selective IgA deficiency (SIgAD) [51,52]	periodontitis, hyperplastic candidal infection, stomatitis, aphthae, herpes labialis, oral ulcerations, most studies show normal periodontal condition.	All ages, usually childhood	Unknown
Transient hypogammaglobulinemia of infancy (THI) [53]	oral candidiasis	Infancy, childhood	Unknown
X-linked agammaglobulinemia (XLA) [54]	necrotizing stomatitis, gingivitis, candidiasis, recurrent aphthae, possible septicemia from odontogenic infection.	Childhood	XL
**DISEASES OF IMMUNE DYSREGULATION**			
APECED (APS-1), autoimmune polyendocrinopathy with candidiasis and ectodermal dystrophy [55]	dental enamel hypoplasia, chronic candidiasis.	Childhood adolescent,	AR
Chediak–Higashi syndrome [56]	early-onset aggressive periodontitis, extensive loss of alveolar bone leading to tooth exfoliation, increased tendency for postoperative bleeding, severe gingivitis, ulcerations on the buccal mucosa, the tongue, and hard palate, candidiasis.	Childhood	AR
Griscelli syndrome type 2 (GS2) [57]	periodontal disease, oral ulcers.	Childhood, infancy	AR
**CONGENITAL DEFECTS OF PHAGOCYTE NUMBER OR FUNCTION**			
Barth syndrome or 3-methylglutaconic aciduria type II [58]	oral ulcers.	Childhood	XL
Chronic granulomatous disease (CGD) [59]	recurrent aphthous-like ulceration, gingivitis, periodontal inflammation, prepubertal periodontitis, ↑ fungal infection.	Infancy, childhood, adolescent, adult	AR/XL
Clericuzio syndrome (poikiloderma with neutropenia) [60]	delayed dental eruption.	Neonatal, infancy, childhood	AR
Cohen syndrome [61]	oral ulcers, teeth agenesis.	Antenatal, neonatal, infancy	AR
Cyclic neutropenia [62]	mucosal ulcers, gingivitis, periodontal disease, aphthous lesions.	All ages	AR
GATA2 deficiency [63]	oral lesions caused by HPV, oral ulcers and blistering, gingival hyperplasia, gingivitis.	Adult	AD
Glycogen storage disease type 1b [64]	hyperplastic/hypertrophic gingiva, giant cell granulomatous epulis, oral ulcers (aphthous gingivostomatitis), delayed dental development and eruption, enamel hypomineralization, oral bleeding, ↑ caries.	Neonatal, infancy	AR
JAGN1 deficiency (SCN6) [65]	dental malformations, amelogenesis imperfecta.	Neonatal, infancy	AR
Kostmann syndrome [66]	severe periodontitis with extensive bone loss, desquamative gingivitis, ulcers, abscesses, candidiasis.	Neonatal	AR
Leukocyte adhesion deficiency type 1–3 (LAD1-3) [67]	severe gingivitis, periodontitis, early tooth loss, persistent oral ulcers.	Infancy, childhood	AR
Localized juvenile periodontitis [68]	periodontitis only.	Adolescent	AR
Papillon–Lefèvre syndrome [69]	early severe destructive periodontitis (primary, permanent)	Neonatal, infancy, childhood	AR
Severe congenital neutropenias (SCN 1) [70]	recurrent oral ulcers, periodontal diseases.	Childhood	AD
Shwachman–Diamond syndrome (SDS) [71]	delayed dental development, oral ulcers.	Antenatal, neonatal, infancy, childhood	AR
SMARCD2 deficiency or specific granule deficiency 2 (SGD2) [72]	malposed teeth, enamel hypoplasia.	Neonatal, infancy	AR
VPS45 deficiency (SCN5) [73]	oral candidiasis.	Neonatal, infancy	AR
WDR1 deficiency [74,75,76]	severe aphthous stomatitis, oral candidiasis.	Infancy, childhood	AR
**DEFECTS IN INTRINSIC AND INNATE IMMUNITY**			
Chronic mucocutaneous candidiasis (CMC) [77]	mucocutaneous viral infections e.g., HSV-1 gingivostomatitis, oral squamous cell carcinoma, dental abnormalities (peg-shaped incisors), delayed exfoliation of primary molars, enamel erosion.	Infancy, childhood	AD/AR
CARD9 deficiency [78,79]	oral candidiasis.	All ages	AR
CD16 deficiency [80]	HSV-1 gingivostomatitis.	Infancy, neonatal, childhood	AR
Germline mutations affecting interferon (IFN) signaling pathway [81]	HSV-1 gingivostomatitis.	All ages	AD/AR/LOF
IRAK-4 and MyD88 deficiencies [81,82]	periodontal bacterial infections, oral candidiasis.	Childhood	AR
Mendelian susceptibility to mycobacterial disease (MSMD) [83]	chronic mucocutaneous candidiasis, anodontia or oligodontia.	All ages	AD/AR/X
Predisposition to mucocutaneous candidiasis [77,81]	recurrent/persistent and/or severe oral mucosal candidiasis.	Infancy, childhood	AD/AR
WHIM syndrome (warts, hypogammaglobulinemia, infections, myelokathexis) syndrome [84]	oral warts, HPV-related oral squamous cell carcinomas, early-onset periodontitis with early tooth loss.	Childhood, adolescent, adult	AD/GOF
**AUTOINFLAMMATORY DISORDERS**			
CANDLE syndrome [85]	severe aggressive periodontitis.	Neonatal, infancy	AD/AR
Cherubism [86]	maxillofacial bone degeneration, early loss of teeth, failed eruption of teeth.	Childhood	AD/AR
Familial Mediterranean fever (FMF) [87]	frequent oral ulcerations.	Infancy, childhood, adolescent, adult	AR/LOF
Mevalonate kinase deficiency (MKD) (hyper IgD syndrome) [88,89]	oral ulcerations.	Infancy	AR
PFAPA syndrome [90]	frequent aphthous-like lesions.	Infancy, childhood	Unknown
SAMHD1 deficiency [91]	oral ulcerations.	Neonatal, infancy	AR
**COMPLEMENT DEFICIENCIES**			
C1 inhibitor deficiency [92,93]	hereditary angioedema. (a case of swelling localized only to the gingiva was described)	Childhood	AR
C3 deficiency [94]	↑ infection with encapsulated microbes.	Childhood	AR
Partial C4 gene deficiencies [95]	periodontitis.	Childhood?	AR
C5 single nucleotide polymorphism (rs17611) [96,97]	periodontitis.	All ages	Co-dominant
**BONE MARROW FAILURE**			
Dyskeratosis congenita [98]	leukoplakia, early-onset aggressive periodontitis, taurodontism.	Neonatal, infancy, childhood, adolescent, adult	AD/AR/XL
Fanconi anemia [99]	increased tendency to caries, aggressive periodontitis, additional teeth, congenital absence of teeth.	Childhood	AR/XL
**PHENOCOPIES OF INBORN ERRORS OF IMMUNITY**			
Chronic mucocutaneous candidiasis (CMC) [97]	long-standing multiple mucosal and tongue fungal infection.	Infancy, childhood	AD/AR
Good syndrome [100,101]	lichenoid-like lesions.	Adult	Unknown

**Table 4:** Diagnostic scheme for inborn errors of immunity.

**Medical Interview**	
➢ Recurrent/prolonged/chronic/opportunistic/unusual infections ➢ Autoimmunity ➢ Hematological disorders ➢ Tumors ➢ Allergies (unusual and/or difficult to treat)	
**RULE OUT SECONDARY IMMUNODEFICIENCIES**	
**ANTIBODY PRODUCTION DISORDERS SUSPECTED**	
➢ **IF POSSIBLE PERFORM:**	
Morphology with manual smear	Assesses the number of WBCs and lymphocytes Antibodies (i.e., immunoglobulins) are produced only by B lymphocytes!
Proteinogram with evaluation of total protein and γ-globulin fraction	Can detect protein loss and secondary antibody deficiency Immunoglobulins are mainly contained in the γ-globulin fraction (all immunoglobulin classes) and to a small extent in the β-globulin fraction (IgA and IgM)
Determine concentrations of major immunoglobulin classes IgA, IgG, IgM	The tests available at any commercial laboratory and most hospitals The cost of the test is relatively modest in comparison to other specialized tests and examinations that patients may perform independently It is crucial to note the distinct norms that pertain to specific age groups
Lymphocyte phenotyping	Allows for the determination of the number of B lymphocytes
ANTIBODY PRODUCTION DISORDERS SUSPECTED ➢ REFER THE PATIENT TO AN IMMUNOLOGY CLINIC	
**CELLULAR IMMUNE DISORDERS SUSPECTED**	
➢ **IF POSSIBLE PERFORM:**	
Morphology with manual smear	➢ Assess lymphocyte count
Lymphocyte phenotyping	➢ Allows you to determine the number of T lymphocytes (T, Th, Tc)
CELLULAR IMMUNE DISORDERS SUSPECTED ➢ REFER THE PATIENT TO AN IMMUNOLOGY CLINIC	
**PHAGOCYTOSIS DISORDERS SUSPECTED**	
➢ **IF POSSIBLE PERFORM:**	
Morphology with manual smear	Assess neutrophil count
Phagotest and Bursttest	Assess respiratory burst
PHAGOCYTOSIS DISORDERS SUSPECTED ➢ REFER THE PATIENT TO AN IMMUNOLOGY CLINIC	
**DISORDERS OF THE COMPLEMENT SYSTEM SUSPECTED**	
➢ **IF POSSIBLE PERFORM:**	
CH50, C3, C4	Determine the functional status of the classical complement pathway
AH50	Determine the status of the alternative complement pathway
DISORDERS OF THE COMPLEMENT SYSTEM SUSPECTED ➢ REFER THE PATIENT TO AN IMMUNOLOGY CLINIC	

**Table 5:** IEIs result in tooth loss.

Inborn Error of Immunity	Treatment Considerations
**Note:** Classic mechanical cleaning (if necessary, under general anesthesia), repeated oral hygiene instructions, mouth rinsing with, e.g., chlorhexidine, extraction of seriously affected primary and/or permanent teeth, and close and frequent follow-up visits are recommended for all patients.	
Chediak–Higashi syndrome (CHS) [56,134,135,136,200]	there is no definitive treatment to stop the resorption of alveolar bone and premature tooth loss conventional periodontal treatment, and systemic antibiotics are recommended immune status and transplantation influence the timing and necessary precautions (e.g., use of antibiotics) management of complications should focus on supportive care, such as using antibiotics to treat bacterial infections aggressive recurrent periodontitis may not respond effectively to scaling and root planning or antibiotic therapy for extractions or surgeries, consider additional methods for achieving hemostasis and avoid NSAIDs due to potential platelet dysfunction prosthetic treatment for missing teeth may be considered based on the patient’s overall medical condition there is no causal treatment for the disease; therapy includes the administration of drugs, including antibiotics in the event of infection, as well as transfusion of red blood cell concentrate or platelets in periods of anemia, and bone marrow transplantation is also possible
Chronic granulomatous disease (CGD) [59,201]	recommended early diagnosis and aggressive treatment of infections and appropriate surgical debridement of localized disease prophylactic use of antibiotics and antifungal drugs the most important element of long-term treatment in non-transplanted patients exogenous IFN-gamma oral glucocorticotherapy—treatment of inflammatory manifestations of CGD allogeneic hematopoietic cell transplantation (allo-HCT)—the only method of CGD therapy that offers a chance for permanent cure
Cherubism [199,202,203]	in most cases, the course is uncomplicated the disease progresses until puberty, and then begins to subside milder cases usually remain under observation in the case of more advanced changes, surgical treatment is considered, consisting of removing the affected bone fragments and, if necessary, replacing them with autografts from the iliac plate; the surgery must be planned very carefully because the facial area is very well innerve, ted and nerve damage can lead to impaired facial expression and sensation; it is performed only after reaching puberty calcitonin therapy malocclusions resulting from cherubism should be treated orthodontically only after the disease has stabilized, i.e., after puberty; it is possible to include impacted permanent teeth in the bite
Cyclic neutropenia (CyN) [62,204]	Granulocyte-Colony Stimulating Factor (G-CSF) is used in the treatment in the lowest dosage ensuring ANC > 500–1000/µL in selected cases, antibiotic prophylaxis is used in exceptional cases, allo-HCT is used
Leukocyte adhesion deficiency type 1 (LAD1) [67,200,205]	periodontal disease may be resistant to nonsurgical periodontal treatment and strict home care routines immediate, targeted antibiotic therapy is recommended additional treatments may involve granulocyte/thrombocyte transfusions, recombinant factor VIIa, and intravenous immunoglobulins prophylactic antibiotics should be administered before dental procedures administration of anti-IL12/IL23 monoclonal antibody—ustekinumab in severe cases—HCT
Papillon–Lefèvre Syndrome (PLS) [69,200,206]	the treatment of oral manifestations is determined by the patient’s age, psychological condition, and tooth mobility it may involve nonsurgical approaches, such as monthly scaling and root planing and systemic antibiotics, and/or the extraction of non-salvageable teeth. alveolar bone loss can make prosthetic rehabilitation difficult
WHIM Syndrome (Warts, Hypogammaglobulinemia, Infections, Myelokathexis) syndrome [84,206,207]	Granulocyte-Colony Stimulating Factor (G-CSF) is used in the treatment in the lowest dosage ensuring ANC > 500–1000/µL in selected cases, antibiotic prophylaxis is used in exceptional cases, allo-HCT is used

## Data Availability

No new data were created or analyzed in this study. Data sharing is not applicable to this article.
